# Supplementary material for: Yiai Fuzheng decoction inhibits triple-negative breast cancer by remodeling the immune microenvironment
Source: Front Immunol. 2025 Sep 30;16:1615631. doi: 10.3389/fimmu.2025.1615631 (PMC12518410; doi:10.3389/fimmu.2025.1615631)
Supplement: Supplementary file 2 [file DataSheet1.zip › Supplementary File 2/Treatment/D_vs_H/07_Pathway_Analysis/Pathway_Analysis/Sig_Pathways_ID/Aminoacyl-tRNA biosynthesis.pdf]

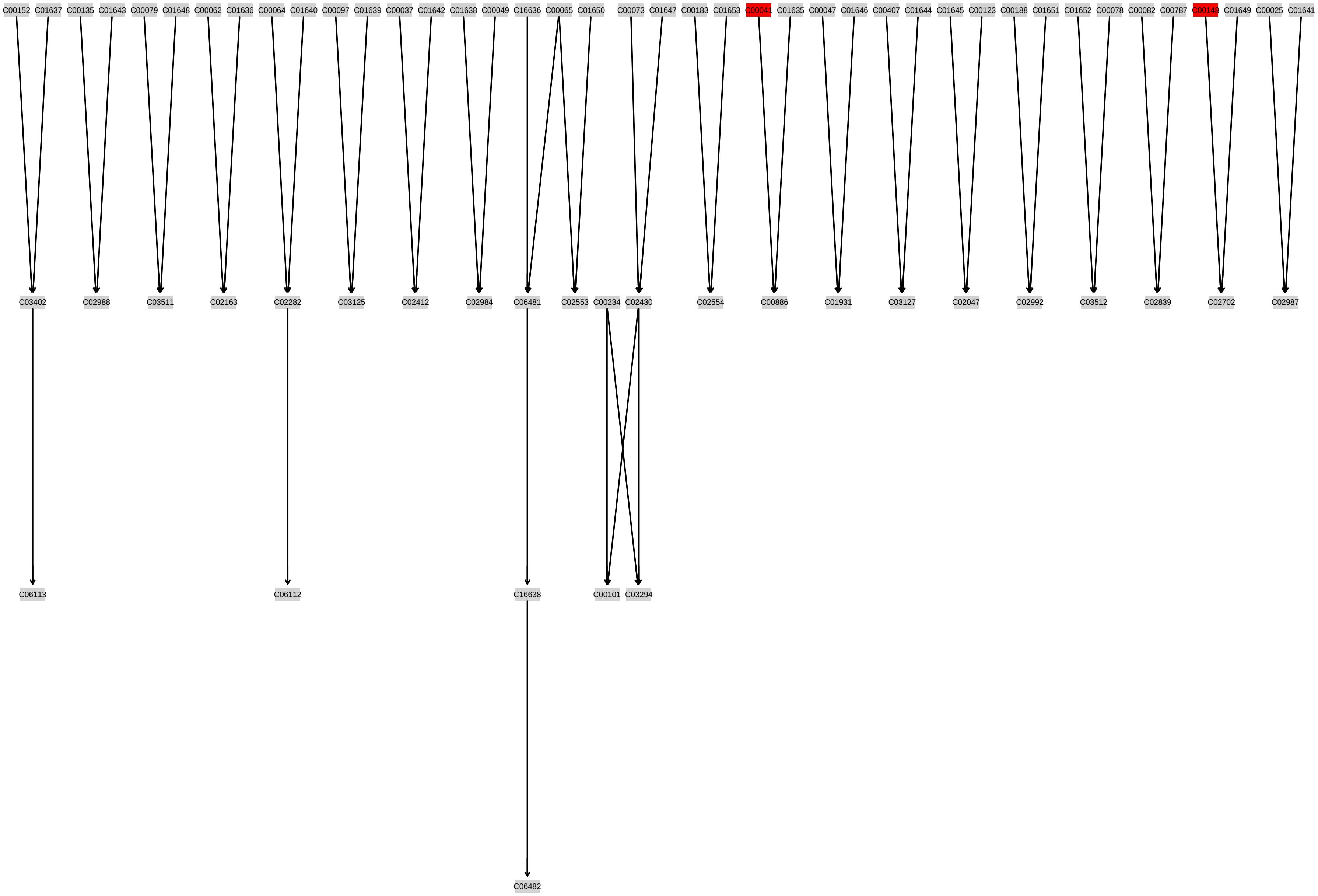
C00152
C01637
C00135
C01643
C00079
C01648
C00062
C01636
C00064
C01640
C00097
C01639
C00037
C01642
C01638
C00049
C16636
C00065
C01650
C00073
C01647
C00183
C01653
C00041
C01635
C00047
C01646
C00407
C01644
C01645
C00123
C00188
C01651
C01652
C00078
C00082
C00787
C00148
C01649
C00025
C01641
C03402
C02988
C03511
C02163
C02282
C03125
C02412
C02984
C06481
C02553
C00234
C02430
C02554
C00886
C01931
C03127
C02047
C02992
C03512
C02839
C02702
C02987
C06113
C06112
C16638
C00101
C03294
C06482
